# Executive Decision-Making: Piloting Project ECHO® to Integrate Care in Queensland

**DOI:** 10.5334/ijic.5512

**Published:** 2020-12-04

**Authors:** Perrin Moss, Nicole Hartley, Jenny Ziviani, Dana Newcomb, Trevor Russell

**Affiliations:** 1 Children’s Health Queensland, South Brisbane, Queensland, AU; 2 The University of Queensland, South Brisbane, Queensland, AU; 3 School of Business, The University of Queensland, St Lucia, Queensland, AU; 4 School of Health and Rehabilitation Sciences, The University of Queensland, St Lucia, Queensland, AU

**Keywords:** Project ECHO, intrapreneurship, integrated care, investment, paediatrics, decision-making

## Abstract

**Introduction:**

A Queensland project team secured grant funding to pilot Project ECHO®, a telementoring model, to drive vertical and horizontal integration across paediatric, education and primary care services. This study sought to understand what influenced healthcare executives’ decision-making processes to organisationally commit to and financially invest in the pilot proposal within an organisational context.

**Theory and Methods:**

A phenomenological approach methodology was adopted to investigate healthcare executives’ conscious decision-making processes. Semi-structured interviews with key stakeholders were conducted alongside project documentation analyses to create a thematic framework.

**Results:**

The qualitative thematic analysis identified five key themes that influenced the decision-making processes of healthcare executives to invest in Project ECHO® as an integrated care pilot. The themes were: (i) personal experiences, (ii) benefits, (iii) risks, (iv) partnerships, and (v) timing. Executives’ reflections explored how their decision-making processes considered the intrapreneurial project team as an indicator of future sustainability.

**Discussion:**

Findings highlighted healthcare intrapreneurs’ drive to foster more integrated and people-centred approaches to care. Intrapreneurial aims of financial sustainability, ongoing improvement and scalability of the proposal positively influenced investment confidence.

**Conclusion:**

Intrapreneurial champions must provide a compelling narrative to convince executive decision-makers that benefits will outweigh risks, that integration is achievable through strengthened partnerships as well as future sustainability beyond the pilot phase.

## Introduction

In 2016, the Queensland (Australia) state government invested AU$35 million in an Integrated Care Innovation Fund (ICIF) to support integrated responses to healthcare, recognising that the system needed to innovate in response to evolving needs of the community [1]. The Queensland Minister for Health and Minister for Ambulance Services launched the fund to stimulate collaborative integrated care proposals from Hospital and Health Services (HHSs) to partner with Primary Health Networks (PHNs) across Queensland. HHSs are state-based and funded secondary and tertiary health providers, and PHNs are nationally funded organisations with commissioning responsibilities to improve the efficiency and effectiveness of primary care services for local communities [2]. Both HHSs and PHNs are governed by boards of directors and funded through service agreements with state and federal government departments. The ICIF approach aligned with international health reform initiatives to drive sustainability and support mechanisms for effective integrated primary-secondary health governance models [1,3].

The ICIF initiative also responded to the need in Queensland for government investment to innovate models of care that would support patient flow across the health system, delivering efficient, high-quality healthcare closer to home [1,3,4]. Key eligibility criteria for applications included that proposals had to demonstrate capacity to be scalable, replicable and sustainable beyond the grant funding period of two financial years (2016–17 and 2017–18) [4]. The ICIF sought applications to implement new ways of working which delivered better integration of care; addressed fragmentation in services; and provided high-value healthcare [4].

A project team led by a General Practitioner Liaison Officer (co-author 4) and a Project Manager (author 1) from Children’s Health Queensland (CHQ), the state’s tertiary paediatric HHS, applied for and was successful in obtaining an ICIF grant for AU$1.1 million. The grant sought to implement and pilot Project ECHO® (Extension for Community Healthcare Outcomes) [5] in Queensland to support General Practitioners (GPs) to manage children with stable Attention Deficit Hyperactivity Disorder (ADHD).

This grant application was endorsed for submission by CHQ’s executive leadership team and assessed by a Selection Committee of independent healthcare and academic executives convened by the Queensland Department of Health [1]. These internal and external executives were critical decision-makers tasked with assessing all ICIF applications against strategic criteria of merit that focussed on integrating healthcare in Queensland.

Project ECHO® is a telementoring model of care which harnesses videoconferencing technology to link primary care clinicians (e.g. General Practitioners, Nurse Practitioners, Practice Nurses, Psychologists, etc) and other frontline service providers (e.g. Teachers, Guidance Officers, Child Safety Officers, Police Officers) in underserved and/or disadvantaged communities (spoke sites) with interprofessional panels of content experts [5]. These content experts (paediatricians, nursing, allied health, educators, consumer representatives) are typically based at metropolitan tertiary health centres (hubs) delivering virtual ‘teleECHO™’ clinic sessions [5]. These clinics run regularly and involve brief educational lectures and case-based, experiential learning facilitated via videoconference sessions known as telementoring series.

The objective of the ICIF funding application was to establish and resource a Project ECHO® hub at CHQ’s Centre for Children’s Health Research (CCHR) in South Brisbane, Queensland, employing hub operational staff, and launching a pilot telementoring series. This series of teleECHO™ sessions would support primary care providers in managing children with stable ADHD locally throughout Queensland with advice and mentorship from a Brisbane-based virtual panel consisting of content experts including paediatricians, educators and parent representatives. Beyond the two-year grant term, the project team sought to acquire additional grant investment to generate own-source revenue through commissioning arrangements to deliver additional teleECHO™ series to spoke participants nationally. This was aimed at transitioning the pilot into a sustainable business as usual operation.

Given the significance of the success of the initial grant funding support for the integrated care initiative, this study sought to explore and identify the organisational, personnel and environmental factors which influenced Queensland healthcare executives’ initial decision-making process to organisationally commit to and financially invest in piloting this telementoring model to deliver integrated care. At the time of the ICIF funding application, the ECHO model™ was untested within the Queensland context, which presented an element of financial risk to the organisation in piloting the model.

This study investigated the decision-making processes of key healthcare executive decision-makers from across the Queensland Department of Health (as the system administrator and ICIF grant funder), one secondary and tertiary healthcare service (CHQ, as the provider and piloting organisation) and two PHNs (as primary care service commissioning agencies, and pilot partners). These executives all played key roles in the decision-making process for evaluating, endorsing and approving the pilot proposal for funding [1].

This study utilised a qualitative approach, using a phenomenological perspective in conducting semi-structured interviews. A total of eight key healthcare executives that were involved in the ICIF grant decision to invest in and pilot Project ECHO® in Queensland were interviewed. A thematic framework was developed to analyse the findings.

### Why are healthcare executive decision-making processes important?

While health systems are susceptible to economic pressures globally [1,3,6], they are environments that are prone to continuous transformational change. There is currently no published research that explores healthcare executives’ decision-making processes regarding investment and sustainability indicators of integrated care pilots such as the Project ECHO® example in Queensland.

While Project ECHO®’s alignment to key learning theories has been well-documented in North America [7], the analysis has been from the perspective of healthcare providers participating in teleECHO™ sessions, rather than the decision-making processes of healthcare executives endorsing the model to be piloted within an organisational context. The CHQ project team’s proposal sought to pilot and sustain Project ECHO® in Queensland by creating an autonomous, self-funding, opportunity-driven service model which could dynamically contribute to reforming the healthcare system. The findings of this study aim to address this gap in knowledge so other project teams can better understand how healthcare executives make investment decisions in the current healthcare system context.

### What is Project ECHO®? Integrated Care through Telementoring

Project ECHO® is a model which can be used to create virtual knowledge networks, or communities of practice, which incorporate case-based learning strategies from medical education and theoretical frameworks that include Social Cognitive Theory, Situated Learning Theory, and Community of Practice Theory [7]. The ECHO model™ was developed in 2003 by Professor Sanjeev Arora at the University of New Mexico (UNM) in the United States, as a platform for both improving healthcare service delivery and patient outcomes in treating Hepatitis C [8]. In 2011, UNM demonstrated that Project ECHO® supported primary care providers to achieve equitable health outcomes in managing patients with Hepatitis C as those treated exclusively in tertiary hospital settings [8]. It was highlighted that where geography prevented equitable access to high-quality care, in particular specialist care, the ECHO model™ overcame this barrier by connecting rural and remote providers with metropolitan-based experts [8]. Thus, Project ECHO® achieved positive health outcomes for patients accessing enhanced healthcare services conveniently in their local communities [3,4,8].

Project ECHO® is a learner-centric virtual hub-and-spoke model of education, based on the principles of “all teach and all learn” [9]. Specialist teams at the ‘hub’ mentor primary care and frontline providers, including General Practitioners (GPs), educators and other health/human services professionals at ‘spokes’, and all participants learn from one another’s expertise and insights [9,10]. Spoke participants share their deep knowledge of local social and cultural considerations, and an understanding of realistic approaches to care within their specific communities [4,8]. The specialists offer complementary content expertise, and over time virtual ‘communities of practice’ or ‘knowledge networks’ develop whereby each participant plays a role in co-producing the knowledge and developing the skills to manage complex conditions [7]. While published literature cites gaps and barriers to integrated care in systems and practice, Project ECHO® demonstrates the capability to bring together historically disparate partners [3,5,11]. The ‘Anatomy of an ECHO®’, the telementoring model’s structure, provides a framework for hub experts to facilitate frontline providers presenting their patient cases, asking questions and contributing recommendations [7,9,10]. The primary objective of teleECHO™ sessions is to address the learning objectives of spoke participants, and validate or reframe the group’s contributions of advice and support to foster the sharing of best practices [5,10,11]. TeleECHO™ sessions are distinctly different from traditional telemedicine and webinars. They provide a blend of highly interactive and multi-directional learning between hubs and spokes with real-time learning based on de-identified patient cases [12,13]. Participants are awarded continuing practice development (CPD) points by their relevant professional body after attending a minimum number of teleECHO™ sessions. Participation is free, and participants can return to future telementoring sessions at any time to present previous or new cases for advice.

At the patient level, the ECHO model™ facilitates better access to care at the right time and place [14]. For healthcare providers, the ECHO model™ builds a supportive community of practice over time where capability and capacity grows, resulting in the potential for local management of more patients [14]. From the community level, the ECHO model™ reduces disparities, retains providers in local communities and reduces the need for patients to travel for specialist-level care [5,7,11]. Finally, at a system level, the ECHO model™ has the potential to increase access to best-practice integrated care, improve care quality and cost, and overall system capacity to meet the growing needs of specific populations [3,10].

### The Queensland context – Project ECHO®’s strategic alignment

CHQ’s Project ECHO® ICIF proposal actively sought to democratise knowledge that was centralised in CHQ’s secondary paediatric services in Brisbane, to support the delivery of contemporary, best practice medical care to patients and families across the state. The state of Queensland is over 1.7 million square kilometres in size, which creates a variety of difficulties for people accessing healthcare specialists. These difficulties can be due to factors including remoteness, poverty and cultural barriers. Project ECHO® was thought to provide a platform solution to address the inequity faced by those patients, communities and providers. The principles of Project ECHO® outlined in Table 1 strongly align to elements of other successful integrated models of care delivered at the primary-secondary interface [3,15].

**Table 1:** The Five Principles of the ECHO model™ [5].


A: Amplification: use videoconference technology to leverage scarce resources
B: Share Best Practices: to reduce disparities
C: Case-based learning: to master complexity and increase self-efficacy
D: Web-based database: to monitor outcomes and showcase impact
E: Everyone participates: ‘all teach, all learn’

CHQ was the first organisation to pilot the ECHO model™ to support the paediatric population in Australia. The CHQ project team’s proposal was framed to meet ICIF eligibility criteria and address system gaps in managing children with stable ADHD [15,16] with CHQ’s Integrated Care Strategy [17]. The proposal sought to connect hospital-based sub-specialists, educators and community-based primary care providers across Queensland to enable a more people-centred approach to providing care, as well as horizontal and vertical integration [18,19]. Target providers who joined the telementoring series as spoke participants included general practitioners, educators and other frontline care providers interested in paediatrics from across Queensland.

The project team proposed to establish a governance committee to inform and endorse the implementation of the ECHO model™ at CHQ. Committee representatives included stakeholders from the Department of Health, PHNs, general practice and parent representation. Through this forum it was proposed that executive decision-makers, clinical and non-clinical professionals, and consumers would foster a long-term bond throughout the pilot in key roles as champions, knowledge partners and beneficiaries, similar to the democratic ethos of the ECHO model™ [5,19].

All stakeholders identified to be involved in the project governance committee and project team were encouraged to contribute their personal experiences to co-design creative solutions for how the implementation would meet the needs of patients, families and frontline service providers as a primary objective, as well as the ICIF objectives of integration [1]. The project proposal indicated that it would be critical to maintain stakeholder dialogue throughout the pilot and encouraged a co-design process with parent representatives. This iterative dialogue and rapport building over time between stakeholders illustrated the proposal’s alignment to the principles of the ECHO model™ [1,9].

The ICIF proposal identified how Project ECHO® hub experts and spoke participants could explore where existing local services or support resources (including General Practitioners, Guidance Officers, and Psychologists) could be leveraged to successfully manage patient cases locally. The prospective sessions would then facilitate the scalability of new knowledge and confidence amongst participating primary care providers to support more of their patients locally [7,8,10,11,12,14]. This study explores and identifies the organisational, personnel and environmental factors that influenced healthcare executives’ initial decision to invest in piloting the ECHO model™ to deliver integrated care in Queensland.

## Description of the care practice

### Methods

The investigators used a phenomenological approach to gain an understanding of the decision-making processes of healthcare executives who evaluated and endorsed the initial grant proposal to pilot the Project ECHO® model in Queensland in 2016 [20,21], as well as their subsequent observations and reflections of how their personal and career experiences contributed to their decision-making processes.

A qualitative approach was employed through in-depth interviews with eight key healthcare executive decision-makers. A secondary desktop analysis of supporting project documentation and observational field notes from the interviews was also completed to triangulate the insights gleaned from the in-depth interviews with the eight healthcare executive decision-makers. The selection of interview participants was based on their healthcare executive and decision-making roles, and their involvement in assessing, endorsing and/or providing investment in the pilot implementation of Project ECHO® in Queensland.

Published literature on phenomenological interviewing techniques recommended that the interviewer (project manager, author 1) take detailed observational field notes during the in-depth interviews [21,22]. The purpose of these notes was to capture the interview participant’s body language, intonation, and other cues to better understand the context surrounding their responses, opinions and experiences [21,22]. These notes were treated as field notes.

The author conducted each interview which lasted approximately one hour, and all were hosted in the offices of the participants. Participants were telephoned and emailed to invite them to participate in the research study, with the interview guide, consent form and research information sheet provided to participants prior to the interview being scheduled. All participants were happy to participate. The interviews were voice recorded and transcribed verbatim, sent to participants for member-checking and then analysed by the author in conjunction with observational field notes and project documentary data (grant application criteria, selection committee analysis, grant application, project plan, governance committee minutes and project manager’s implementation diary) that had been collected. The authors then allocated a series of codes which were distilled down to derive the five key themes to analyse the study findings. This process aligned with the descriptive phenomenological human scientific research approach employed in other published studies [21,22,23] and the Consolidated criteria for reporting qualitative research (COREQ) checklist for interviews and focus groups [24].

### Participants

The sample of eight interview participants was the entire stakeholder group of healthcare executive roles who had knowledge of and involvement in the decision-making processes for investing in piloting Project ECHO® in Queensland. A description of the participant demographics is presented in Table 2.

**Table 2:** Demographic data summary of interview participants (N = 8).

Demographics	Participants

Gender	**6** Female (75%) **2** Male (25%)
Professional background	**3** Medical (2 Female, 1 Male) (37.5%) **5** Nursing (4 Female, 1 Male) (62.5%)
Organisational responsibilities	**5** Director-General/Chief Executive/Executive Director equivalent with strategic, financial and inter-agency accountabilities (62.5%) Participants: 1, 2, 3, 4, and 7. **3** Clinical/Academic Director equivalent with operational, research and practice improvement accountabilities (37.5%) Participants 5, 6, and 8.
Education	100% had a postgraduate qualification
Regional/Rural/Remote Experience	**4** (50%) had healthcare executive experience operating outside of a metropolitan centre
Primary Care Experience	**5** (62.5%) had work experience in the primary care sector

### Ethics Statement

The CHQ and University of Queensland Human Research Ethics Committees approved this study under reference number: LNR/18/QCHQ/44762.

### Data analysis

The investigators were confident that the total sample of eight interview participants achieved data saturation for coding and meaning as this was the entire representation of the total population [25,26]. The interview transcripts were analysed using NVivo 12, a qualitative analysis software program [27]. A thematic framework and coding guide were developed during the analysis process. Data was coded according to key themes.

Inter-rater reliability was achieved with the data by three of the co-researchers to ensure that a consensus on the thematic coding was achieved. The research team reviewed the qualitative data to describe the themes that impacted healthcare executives’ -decision-making processes to organisationally commit to and financially invest in the Project ECHO® pilot. These themes allowed for the data to be distilled during the reduction process [21]. The observational notes and secondary data sources were utilised to validate references made by interview participants during the primary interviews and contextualise the broader eco-system in which their investment decisions were being made. Key points of reference in observational notes and secondary data sources were coded using the same coding list as the interview transcripts for consistency.

### Results

During the thematic analysis, five key themes were identified that informed the decision-making processes of healthcare executives organisationally committing to and financially investing in the pilot. These themes were (i) personal experiences, (ii) benefits, (iii) risks, (iv) partnerships, and (v) timing. To describe the themes that enhanced the executives’ decision-making in favour of investing in and piloting Project ECHO® in Queensland, elements that cemented their decision-making are illustrated as facilitators and barriers, supported with direct quotations in Table 3.

**Table 3:** Thematic framework for the Project ECHO® innovation that influenced healthcare executives’ decision-making.

Emerging Themes	Facilitators	Barriers	Quotes

**(i) Personal Experiences:** Proposal appealed to healthcare executive’s career journey, personal values and motivations;	Executive had rural and remote working experience and lived experience with frontline challenges Executive’s personal values were conducive to innovation agenda and organisational values Executive saw innovation as essential driver for ongoing system improvement Executive was supportive of testing new solutions to system problems Executive had international work experience Executive identified personal commitment to champion Project ECHO® proposal within their role Executive had knowledge of project team’s strong track record Executive identified characteristics of the project team that were consistent with intrapreneurship [6,29,30,31,33,34,35,36,37,38,39]	Executives that did not have rural and remote working experience	**Career Journey:** “Having grown up in a different culture, I think that that has shaped me. Then having trained in a clinical discipline, and having worked across a number of different clinical areas, that has shaped my decision making.” – Participant 4 “My experience working with parts of our communities who are disadvantaged, in particular, really has heightened my awareness and appreciation of looking at anything that can make a positive difference, and a positive impact. And ECHO falls into that category where I can see the potential for it across so many different domains.” – Participant 4 “I think I draw on all of that [experience] whether it’s from being clinical, a doctor, to going into the emergency department setting, and then a rural doctor kind of cradle to grave, with a lot of responsibility 24/7 in a rural area, with very little resources.” – Participant 7 **Personal Values:** “I also use our [organisational] values as a decision-making framework.” – Participant 2 “I feel that our board and our leadership, have an innovative mindset.” – Participant 4 “The concept of ECHO really speaks to how I believe medicine should work. [Medicine] should be more non-hierarchical.” – Participant 6 **Motivation:** “I think that everybody needs to be thinking about innovation. Whether it’s innovation in terms of everyday innovation at the [hospital] bedside, or how we can deliver care in a more streamlined way that improves the parent and family experience and uses resources more appropriately.” – Participant 2 “You can’t take a singular approach and you’ve got to be driven by outcomes for a population or an individual as opposed to retention of power. It requires a more egalitarian approach.” – Participant 2 “Our work is revolving around innovation and change.” – Participant 3 “You’ve got to think differently, and you’ve got to work differently, and take advantage of innovation, of anything that might give you a strategic or business advantage in the market.” – Participant 4 “Yes, it hadn’t been shown in the Queensland context, but [ECHO] was in similar other health systems. So that you could say - you could see that there was a parallel and the organisation feasibility was there.” – Participant 5
**(ii) Benefits:** Proposal created a value-add for ongoing change – innovation was seen to have additional benefits beyond integration (population health outcomes, workforce development, service/quality improvement	Executive noted Project ECHO® proposal clearly articulated objectives of integration and strategic alignment Executive was aware of project team’s experience leading large-scale integration pilots Executive noted Project ECHO® proposal and project team’s potential for financial sustainability and/or self-funding Executive noted value-add: Project ECHO® proposal aligned with organisation’s strategic priorities Executive identified that the Project ECHO® proposal would benefit multiple stakeholders Executive noted Project ECHO® proposal’s primary focus was on patients and frontline workforce Executive noted Project ECHO® proposal showed potential to embed integrated care as business as usual within organisational context Executive saw proposal as a catalyst for change: Project ECHO® had potential to initiate multiple changes and spin-offs within organisational context	Executive identified that workforce development would be a barrier to overcome Paediatricians perceiving GPs as a threat to their professional territory	**Population health outcomes:** “ECHO aligns well with our strategy around workforce development in education, and also integration and innovation in health systems. So, those were the, sort of, three pillars, I suppose, that made ECHO a project that we felt that we could and would support.” – Participant 1 “The opportunity for better integration of our health system, to me, that’s the big opportunity that ECHO provides … it’s that dialogue, that’s the big value …” – Participant 1 “For rural and remote communities where you do need specialist input, and it’s more cost effective to bring it through Telehealth, but still, ECHO has a much more global reach.” – Participant 2 “[ECHO] empowers providers in communities that lack access to care. So obviously that’s a massive benefit for consumers that they could have access to that specialist expertise, wherever they live.” – Participant 6 **Workforce development:** “[Project ECHO] was an opportunity not just to grow GP confidence but to actually encourage some of our own community teams to become involved in developing greater understanding of particular conditions and also could provide an exemplar for how to actually lead co-design with consumers.” – Participant 2 “Project ECHO has been able to be one of those options on the table where we can bring specialists expertise into primary care.” – Participant 3 “Immediately ECHO seemed to me to be something that was exciting from the other side of the fence, as something that is really attractive to be part of, genuinely fun. I think they say that ECHO increases joy of work and professional satisfaction, and all those benefits. And I can see that straightaway.” – Participant 6 “The bottom line is, I will give them money, A) If I’ve got it, but B) If they leave and I have a sense of confidence that they can deliver on it, and it’s worthwhile.” – Participant 7 **Service/Quality Improvement:** “Our health system is increasingly complex, [Organisation is] looking at innovation as a potential solution to some of the challenges we face. I thought there was a lot of potential in the Project ECHO model.” – Participant 1 “Where I look at a proposal it is largely about, low cost, broad impact or if it’s a high cost, then people must be … have sources of funding and have reviewed it with a commercial lens. Lots of people come to the table with a proposal for something that they want to do that’s based on self-interest. So actually … that’s one of my priorities, is to look at any proposal in terms of its broader impact and not support things the individuals simply want to do for themselves.” – Participant 2 “We have opportunities to work much more closely with the community-based sector to deliver innovative models of service delivery, and to do things differently that includes shaping our workforce, models of care, models of service delivery, strengthening the continuum of services.” – Participant 4
**(iii) Risks:** Proposal rated as ‘low-risk, high-reward’ with competitive grant funding available for project lifespan and an autonomous project team with high-calibre track records of success	Executive was comfortable to pilot an innovation that was untested in Queensland Executive saw value in Project ECHO® having an international track-record Executive saw ICIF investment opportunity to pilot Project ECHO® through a non-recurrent grant, rather than at the expense of business as usual operations Executive was comfortable about the project team’s capability to sustain Project ECHO® beyond pilot Executive saw the opportunity to pilot Project ECHO® with grant funding as a safe way to outweigh potential risk of longer-term investment if it did not achieve integration Executive identified that the Project ECHO® proposal anticipated risks had mitigation strategies identified Executive identified that the Governance Committee proposed to leverage necessary experts, advocates and decision-makers to manage risks and maintain strategic direction if the pilot were approved	Executive noted that Project ECHO®’s financial sustainability was always reliant on project team’s capability to self-fund operations Executive highlighted public sector organisations have limited to no recurrent operational funding allocated for innovation Executive acknowledged there was a lack of remuneration for GPs participating in pilot. This kept pilot costs low but presented an ongoing limitation on GP uptake	**Financial Risk:** “The notion that you can seed fund innovation and then somehow it will organically just be built into business as usual, um, often doesn’t work.” – Participant 1 “We need ECHO to be self-funding, in order for it to be valued by the organisation… It needs to become a business, within our business. That’s going to be the critical tipping point.” – Participant 2 “The intention was to fund something as a disruption, and let the system adjust to the new. When you send a financial envelope, what actually tends to happen is that you fund something with one-off disruption money and then in two years’ time, essentially, a service is being created, which then lacks a funding stream.” – Participant 7 **Reward:** “I’ve seen with ECHO, as I’ve seen with other innovation, is the need to go into it with an expectation that how you think it might look at the beginning is likely to be quite different from how it might look at the end.” – Participant 1 “I would expect, you as a leader, that I’ve invested in [developing] to tell me what you’re learning about, and bring that to the executive and board table and provide [us with] guidance.” – Participant 2 “If people are following and believing in the vision, underpinned by a set of values, I think we can do anything.” – Participant 2 “I saw the immediate scalability of the model because it was so simple, but, in its simplicity, very well constructed.” – Participant 4 “When I looked a little bit deeper and saw the impact, then that confirmed for me the efficacy of the approach and the potential that it had.” – Participant 4 “It’s important to have lived experience and to have had variety in your career around success and failure. And for me, the failures in my life and in my career, have always taught me more than the victories.” – Participant 4 “It’s allowing you to have the space to develop those ideas and have a voice, and be listened to.” – Participant 5
**(iv) Partnerships:** Proposal demonstrated capacity to integrate with internal and cross-sector agencies	Executive reflected that they trusted the project team to pursue partnerships autonomously Executive felt that the Project ECHO® proposal would create new integrated, non-traditional partnerships Executive identified the Project ECHO® proposal’s potential partnership opportunities across vertical and horizontal stakeholders both internally and externally to the organisation Executive identified project team’s commitment to engaging consumers in the project governance structure to ensure their voices and expertise could be harnessed in the co-production process Executive noted the Project ECHO® proposal sought to develop and leverage known partners to capitalise on existing working relationships to achieve milestones Executive identified that the Project ECHO® proposal would foster a shared risk and reward governance approach amongst partners during pilot Executive saw value in the Project ECHO® proposal to integrate systems by connecting providers using new technologies	Executive identified that working in partnership with other agencies would include juggling competing priorities	**Internal Partnerships:** “We have administered the ECHO at a scale which could be capitalised on to actually do more with less moving forward. And there’s no reason why we couldn’t use this for the adult population as well.” – Participant 2 **Cross-Sector Agency Partnerships:** “We thought it definitely would be interesting to see how it [the pilot] was received because at that point we weren’t able to anticipate how our GPs would receive ECHO.” – Participant 3 “We need to look for investors now to partner with us, and we need to bring our pitch to that table, to look for an investor that can bring us to scale and in potentially different markets.” – Participant 4 “I thought it was a well-developed proposal. I thought it was one of the few that had clearly, put significant effort into working with the community sector and general practice sector.” – Participant 8 “We’re in here boots and all and we are really going to make sure that all the community groups are really linked in. So that was very reassuring.” – Participant 8
**(v) Timing:** Proposal leveraged point-in-time policy, investment, workforce and reputational opportunities	Executive saw opportunity in Project ECHO® proposal to acquire significant government investment to pilot the innovation Executive identified that their role was important to socialise and advocate for integrated care theory and practice in the organisational context Executive identified they had an appetite for innovation to improve outcomes within the organisation Executive had confidence that the project team could mobilise operations rapidly if successful in acquiring funding, and would remain agile to ongoing opportunities Executive had confidence in the project team’s proposal to attract future investment beyond the ICIF grant term	Nil identified	**Policy:** “There are some innovations that just shift thinking within the system… it could deliver a shift in thinking for another business case and leverage a larger innovation or contribute to a larger body of work. So, I’m always thinking about, are there opportunities to join the dots up between different pieces of work?” – Participant 4 “Are those ideas that are ahead of their time? It’s important not to dismiss those because the timing just isn’t right.” – Participant 4 **Investment:** “I saw this as an opportunity to get some money and develop a … more of a knowledge base around ECHO and grow that competence.” – Participant 2 “In an environment like this, when there is a drive for efficiency, I think, initiatives like ECHO can be at risk or can come into their own.” – Participant 4 **Workforce:** “I still make mistakes around decisions that I make. But I learned from those and I tried to balance that out by failing fast. So, stay close to the work initially, stay close to the decision, completely back it, sponsor it, encourage those who own it. Hold them to account. But if I consider that it’s not working, I simply won’t let it limp through. I’d rather fail fast, stop, look at the learnings, and move on.” – Participant 4 **Reputational:** “We have a sustainability strategy that’s not just about financial sustainability, it’s about all the other aspects of how we do business that will influence our sustainability as a leader.” – Participant 2

These themes also linked to how executives’ personal experiences and career goals/motives positioned them to consider innovation pilots as vehicles to drive workforce and systems performance and productivity to benefit patients and communities [28,29,30]. For ease of analysis, where the interview participant recognised a facilitator or a barrier to their decision-making, these were recorded to inform the theming.

The results indicated that where there was strong support of the Project ECHO® pilot, healthcare executives had referenced their own regional/rural/remote work experience. References to project team’s leadership, stakeholder engagement and indicators of financial sustainability for this innovation were identified as critical factors in decision-making across all themes. The healthcare executives noted that the credentials, track record and combined characteristics (experience, drive, autonomy, technical and professional expertise) of the project team, paired with indicators of sustainability gave them confidence to invest in the proposal [6,31,32]. In this context, the project team’s intent and aim to develop a financially sustainable approach was a key influence in the executives’ decision-making process which had potential to be replicated across other improvement initiatives and business as usual operations.

## Discussion

### CHQ’s Project ECHO® proposal: using intrapreneurship to pilot integrated care

Healthcare executives, by the nature of their roles, often face challenging decisions. As identified in this research, executives’ decision-making is informed by their (i) personal experiences, and ability to analyse (ii) benefits, (iii) risks, (iv) partnerships, and (v) timing of events within organisational and system contexts to invest in pilots seeking to integrate care.

In the case of personal experiences, this study found synergy in the executive’s career journey, personal values and organisational motivations as clear indicators of whether they would have invested in the Project ECHO® proposal. Each executive that had rural and remote work experience identified with the perceived benefits and scalability of Project ECHO® to achieve integration beyond a metropolitan context and empower local communities. Hence, an executive’s rural and remote work experience provided a direct association with the fundamental mechanisms embodied in the integrated care project to be funded, and were seen as a strong driver of support. Where executive decision-makers did not have rural and remote work experience, they were still able to anticipate benefits and partnerships for spoke participants in rural and remote settings. This lack of first-hand experience working in rural and remote settings did not have an unfavourable influence on their decision-making to endorse the proposal.

Further, healthcare executives identified perceived benefits they associated with the Project ECHO® proposal beyond achieving vertical and horizontal integration as an influencing factor in their decision-making. Namely, they saw Project ECHO® serving as a catalyst to redesign existing services to yield greater impact and efficiency, to enhance workforce capability amidst growing fiscal pressures, and improve service/quality outcomes at an organisational and system level. These themes were closely aligned to the ICIF grant’s scalability criteria to achieve integration.

This contributes a unique insight into how healthcare executives consider the potential for proposals to be a change agent for scalable and sustainable improvements elsewhere within the organisational context. While the healthcare executives remained pragmatic of the success rate of innovative pilots in general [6,31,32,38,39], their interest in Project ECHO®’s future potential and trust in the project team outweighed their caution and aligned to the characteristics of intrapreneurship [31,33,34,35,36,37,38,39]. The characteristics of the project team, as identified by the healthcare executives, strongly mirrored other published studies on intrapreneurship, whereby motivated individuals employed within established organisations act as change catalysts to adopt, implement and champion change in creative, non-traditional contexts [6,31,32,33,34,35,36,37,38,39].

The risks theme was interesting in the context of the ICIF grant opportunity because the healthcare executives were prepared to test a model that was unknown in the Queensland healthcare system. This was because the executives were comfortable with the ICIF grant securing the investment necessary to test the model, and the proposal’s indications of future sustainability. Despite there being no previous benchmark of Project ECHO® in Queensland, executives sought to use the pilot as a disruptor to stimulate systems thinking around new ways of providing services to meet community need which were fiscally sustainable. Queensland healthcare executives identified that the system gaps and barriers that the ICIF grant opportunity sought to address were consistent with the global literature, and the Project ECHO® proposal carried a sense of assurance that the project team could successfully implement and sustain the pilot [3,31,32,33,34]. Interview participants cited the Project ECHO® proposal’s future financial sustainability as a strong and positive moderator in their decision-making processes, and underwrote the low financial risk. While Project ECHO®’s international reputation was acknowledged to achieve improved health outcomes, it was the intrapreneurial attributes (credentials, track record and combined characteristics of experience, drive, autonomy, technical and professional expertise) of the project team documented in their proposal that also influenced the executive decision-makers’ confidence to invest in the pilot.

Similar to the findings for risks, the drive to forge and leverage partnerships was considered by executives as a strategic opportunity and critical measure of the proposal’s success. Executives saw the Project ECHO® proposal as an opportunity to enhance strategic partnerships and population health outcomes by connecting primary and secondary healthcare service providers virtually, while achieving more cost-efficient workforce utilisation. This research has enhanced the understanding of healthcare executives’ decision-making about Project ECHO®’s potential to broker new partnership opportunities and facilitate workforce transformation over the longer term. The proposal’s governance committee membership also provided assurance to executive decision-makers that the project team could demonstrate commitment to engaging consumers in the pilot implementation. The governance committee structure proposed consumer expertise and influence would be harnessed throughout the implementation phase to enhance co-production processes.

The timing of this proposal was also central in leveraging ICIF grant investment that was made available by the Queensland Department of Health. The project team’s proposal aligned with the ICIF grant criteria and healthcare executive decision-makers’ organisational motivations to utilise this short-term funding opportunity to pilot an internationally renowned model to integrate care.

The themes identified in this research, while focusing on healthcare innovation and investment decision-making, align with other contexts focusing on redesign and improvement more broadly across the public sector [3,19,28,30,40]. In particular, these findings align with public sector organisations empowering motivated individuals through digital innovations, democratising innovation, enabling change, and change-ready business models [19]. In the specific case of the ICIF grant opportunity in Queensland, the findings of this study validate the drive by healthcare executives to implement new models of care, supported by sustainable business models, that show promise of fostering a more integrated, and people-centred approach to care across primary, secondary and tertiary services than what conventional approaches have delivered previously [1,19,28].

Learnings from this study also identified that innovation proposals appeal to executive decision-makers where the project team provides a compelling narrative of how the benefits would outweigh potential low-level risks, strengthen partnerships at the point in time when investment funding is available and consider future sustainability [6,31,33,34,40]. These indicators of what healthcare executives look for in innovation proposals can be generalised to other innovation proposals. These indicators which facilitated executives’ decision-making to support piloting Project ECHO® that were analysed in Table 3 have been generalised in Table 4.

**Table 4:** Benefit Indicators.


1: Innovation proposal had **strategic alignment** and a **clear value-add** to the organisation;
2: Executive decision-makers aware of project team as a **motivated talent pool**;
3: Innovation proposal explored **financial sustainability** beyond the grant term;
4: Proposal **clearly identified beneficiaries and partners** (patients, communities and workforce);
5: Proposal clearly articulated how the innovation would **enable and embed integrated care as a business as usual** function within the organisation;
6: Proposal could serve as a **catalyst for other innovative change** within the organisation.


One limitation of this study was that there were no comparative analyses with other successful ICIF grant projects in Queensland, or unsuccessful applications at that point in time to contrast against the key themes identified in this study. None of the other successful ICIF grant proposals that were awarded in Queensland at the same time investigated healthcare executives’ decision-making processes or Project ECHO® as an innovation prior to implementation. While they were all independently evaluated by an academic institution, the focus was limited to the defined intervention and outcomes, cost consequence/cost effectiveness and economic and implementation outcomes [1]. These project-specific findings were not publicly available.

Specifically, for the Project ECHO® proposal in Queensland, future research is warranted to explore the implementation learnings of comparison sites that have emerged since 2016. Implementation frameworks such as the Project INTEGRATE Framework [41] or the Consolidated Framework for Implementation Research adapted for Project ECHO® [42] could be employed to measure integrated care outcomes of other Australian teams implementing Project ECHO®. Results of these comparisons may identify other project teams employing intrapreneurial approaches to guide, plan, evaluate and sustain operations. These frameworks could enhance reliability of implementation and sustainability data across sites [41,42]. A future comparison study of other Project ECHO® pilots would be useful to demonstrate if the CHQ implementation remained a unique example, and what intrapreneurial characteristics were present in or could be exportable to other contexts.

The investigators used a purposeful sample of eight interview participants that represented healthcare executive roles who would typically be involved in investment decision-making for new innovations in Queensland [25,26]. This analysis represented a moment in time of the decision-making process to invest in piloting Project ECHO® in Queensland. Examples of investment in other Project ECHO® pilot implementations are currently underway in early stages across other locations nationally and internationally which may have different experiences. Sharing the lessons learned at this point from Queensland may help others to better tailor their approach in designing their engagement strategy, proposal and implementation plan to influence executive decision-makers. This would support project teams to attract pilot investment to implement Project ECHO® or other similar integrated care innovations in their own contexts.

By examining the factors that influenced how and why executives made decisions, insights were gained that aligned with recent research in the healthcare setting. Findings of this research highlighted executives self-identifying the value in supporting redesign and improvement, and enabling project teams to innovate [1,31,32,33,34] through exploring integrated and intrapreneurial approaches to innovation in complex care systems. These findings increase understanding of what influenced Queensland healthcare executives to invest in Project ECHO® as an innovation to achieve improved system integration.

The themes, facilitators and barriers identified in this study that gave healthcare executive decision-makers confidence to invest in piloting Project ECHO® in Queensland also aligned with published research about intrapreneurship [6,31,32,33,34]. The concept of intrapreneurship has been defined as individual champions within established organisations that have been legitimised by executive decision-makers, in this case the CHQ project team, who were empowered to mobilise and leverage resources to create new business or service models that could achieve divergent change and challenge the status quo [6,33,34]. These champions illustrated their capability to drive change by mobilising necessary resources including skills, funding and expertise to scale up a discrete innovation to the system level [6,31,32,33,34]. This affirms the value that executive decision-makers place on the credentials, track record and motivations of project teams seeking investment.

This study’s objectives to understand how Project ECHO® was perceived, implemented and could be sustained within an organisational context, and gauge the actual impact on the organisation from the perspectives of healthcare executives were highlighted in Table 3. These themes focused on the personnel, organisational and environmental factors that impacted how they perceived the Project ECHO® proposal would be implemented and sustained by the project team [3,4,6,31,32,33,34]. The attributes demonstrated by the project team in their proposal highlighted alignment with intrapreneurial approaches to source funding, skills, expertise and navigation of a complex, public health organisation to pilot and embed Project ECHO® as an integrated care model [1,6,33,34]. The healthcare executive decision-makers reflected that the proposal and characteristics of the project team were conducive with their analysis of the health system landscape at the time and would be a viable investment of ICIF grant funds [6,33,34].

## Conclusion

Healthcare executive decision-makers operate in a context where demand for services is often exceeding system capacity [1,3]. Innovative and integrated models of care can act as a catalyst for change to improve services and increase workforce capacity available to meet the needs of people in communities. To obtain pilot investment in a competitive, fiscally constrained environment, project teams need to convey their strategic alignment across a number of key focal points. Project teams in the healthcare sector in particular must be able to articulate how their proposal will enhance healthcare service delivery outcomes sustainably.

When assessing innovation proposals, healthcare executives’ decision-making is influenced by a range of factors. Decisions are based around five key themes: (i) personal experiences, (ii) benefits, (iii) risks, (iv) partnerships, and (v) timing. Project teams with intrapreneurial characteristics including their collective experience, drive, autonomy, and expertise [1,6,31,32,33,34] can attract investment from executive decision-makers to pilot and embed new models of integrated care.

This research provides new knowledge about Queensland healthcare executives’ decision-making processes to organisationally commit to and financially invest in pilot proposals to integrate care. This knowledge is valuable to intrapreneurial project teams working in large-scale public sector systems seeking to innovate and integrate care amidst growing fiscal pressures. Findings from this study will inform future proposals to compete for investment opportunities to pilot innovative new models.

This study affirms that project teams need to demonstrate to executive decision-makers that their investment has potential to achieve integration across the continuum and remain financially sustainable beyond pilot phase. By illustrating potential where people-driven care can flourish at scale, executive decision-makers are inclined to support intrapreneurial champions in key project roles that can engage and empower people and communities to reduce inequalities and improve access. This example of ‘integration intrapreneurship’ contributes new evidence which highlights a novel approach to addressing the evolving needs of the community as financial pressures in the healthcare system continue to increase.
